# Geomaterials Evaluation: A New Application of Ashby Plots

**DOI:** 10.3390/ma13112517

**Published:** 2020-05-31

**Authors:** Fuqiang Sun, Ya-Pu Zhao

**Affiliations:** 1 State Key Laboratory of Nonlinear Mechanics, Institute of Mechanics, Chinese Academy of Sciences, Beijing 100190, China; sunfuqiang@imech.ac.cn; 2 School of Engineering Science, University of Chinese Academy of Sciences, Beijing 100049, China

**Keywords:** Ashby plots, coal roof, frictional rupture, landslide, coal-gas outburst, performance index

## Abstract

The development of fossil energy resources and the occurrence of geological hazards call for a quick and effective identification of geological situations. In this study, we present rapid evaluations of geological structures from the pure point of view of material properties. For the first time, Ashby plots are applied to the evaluation of geomaterials such as rocks and coals. A series of case studies are presented and related Ashby plots are drawn. The stability of rocks facing natural hazards is analyzed and compared; the stability of coals formed in different periods in China is studied; and a new brittleness index for reservoir rocks is proposed. The Ashby plots show a strong vitality and a wide application prospect in geomaterial evaluation and geological engineering.

## 1. Introduction

The evaluation of geomaterials such as rocks and soils is essential in geological prospecting, natural hazards prevention, and fossil energy development. A quick, effective evaluation of reservoir rocks can be helpful not only in the design of an exploitation scheme and the promotion of energy productivity [1], but also in the protection of workers’ safety [2] and the mitigation of mining’s impact on the environment [3]. In addition, the evaluation of rock stability against natural hazards such as landslides and earthquakes can give a better understanding of the mechanism and provide a possible prevention strategy for these geological hazards [4,5].

The complexity of geological structures and the heterogeneity of geological masses make it very difficult to evaluate geological materials. There are numerous factors such as functional requirements, geometric parameters, and material properties that must be taken into consideration. Furthermore, they are usually strongly coupled in the evaluation of geological events, i.e., the biosphere, lithosphere, and hydrosphere impacts of hydraulic fracturing [3,6], the deposits’ effects on mineral resources [7], the characterization of reservoir rock properties [8], and the rock permeability in fault zones [4].

In the above situations, one of the challenges is to develop a simple method to simplify the problem and evaluate the geomaterials quickly and effectively. In addition, simple methods are preferred by engineers in the field, since they must quickly identify safe sites for construction and dangerous sites for protection. From this point of view, Ashby plots show strong potential in the evaluation of geomaterials.

Ashby plots have provided a guideline for material selection in engineering applications for decades [9,10]. For those facing complex engineering requirements that involve various factors such as physical properties, technical properties, social benefit, and others, Ashby plots offer a way to quickly evaluate and select the most appropriate material among a large number of available alternative materials. This method is widely used in many fields such as traditional mechanical design [10], microelectromechanical systems (MEMS) [11], constructional engineering [12], additive manufacturing [13], and biomedical engineering [14]. These applications promoted the development of databases for the properties of a large number of materials [15]. Besides the selection of suitable materials, Ashby plots have pointed out the direction of new material design and become the necessary tools to display the high performance of the new materials [16,17,18]. In fact, both the traditional materials and the new materials are all directly derived or indirectly manufactured from natural resources, namely oil for polymers, ores for metals, minerals for ceramics and glasses. However, these geomaterials have not been included in Ashby plots.

In this study, geomaterials such as rocks and coals in natural hazards and fossil energy developments are evaluated using Ashby plots from the pure point of view of material properties. The basic scheme and main procedures are introduced in Section 2. Four geological evaluation examples are given in Section 3 based on physical properties such as density, strength, elasticity, and toughness of rocks and coals. The four cases were selected from representative problems in the development of fossil energies and the prevention of natural hazards, which involved the brittleness of reservoir rocks, the stability of coal roofs and coal walls, the stability of rocks in landslides, and the fracture energy for rocks to form faults. In each case, the key index for the geomaterial evaluation was derived from the given situation, and the relevant Ashby plot was plotted using the data from the literature. Using the performance indices and Ashby plots, the geomaterials were evaluated and compared systematically.

Our aim was to systematically evaluate material properties in natural hazards and to introduce Ashby plots for wide applications in the evaluation of geomaterials, which constitutes the main body of the paper. Concurrently, while evaluating the geomaterials, we systematically showed the stability of the coals formed in different periods in China. A new brittleness index was derived with tensile strength and fracture toughness. The fracture energy of rock joints and interfaces was derived with the shear stiffness and the stress drop in the frictional fractures. The method provides a new idea for evaluating geomaterials in the field. Moreover, the results may help in understanding the material properties connected with natural hazards.

## 2. Materials and Methods

The procedures of geomaterial evaluation with the application of Ashby plots can be divided into the four following main steps [10].

### 2.1. Translation and Metric Derivation

For typical geological cases, the first step is to translate the problems concerned from field expressions into mathematical expressions. The problems concerned include, for example, the brittleness of reservoir rocks, the stability of geological structures, and the maturity of kerogens. In general, the translated mathematical equations can be expressed in the following form:

(1) $$P=f\left[\left(\begin{array}{l} \mathrm{Funtional}\\ \mathrm{requirements},\;F \end{array}\right),\left(\begin{array}{l} \mathrm{Geometric}\\ \mathrm{parameters},\;G \end{array}\right),\left(\begin{array}{l} \mathrm{Material}\\ \mathrm{properties},\;M \end{array}\right)\right]$$

where *P* is the performance metric, which represents the performance of the given geomaterial in a specific case. The performance matric reflects the properties of three aspects, namely the functional requirements, the geometric parameters, and the material properties.

### 2.2. Metric Simplification and Index Derivation

The performance metric can be simplified when the three groups in Equation (1) can be separated and expressed in the following form:

(2) $$P=f_{1}\left(F\right)\cdot f_{2}\left(G\right)\cdot f_{3}\left(M\right),$$

where *f*_1_, *f*_2_, and *f*_3_ are separate functions of functional requirements, geometric parameters and material properties, respectively. This means that the limitations of the structural efficiency coefficient, *f*_1_(F)·*f*_2_(G), can be ignored when one takes only material properties into consideration. That is to say, the performance metric gets its maximum value for all structures when the *f*_3_(M) reaches its maximum. Therefore, the problem is simplified when knowing the expression of *f*_3_(M) and thus *M* becomes the key coefficient named as “performance index”, which can be widely used in geological cases.

### 2.3. Mapping Data

After the performance indices are derived with specific material properties, data gained from field observations or laboratory experiments are plotted in a typical chart. For example, a performance index has the following form:

(3) $$M=\frac{A^{\alpha }}{B^{\beta }},$$

where *A* and *B* are the material properties such as density, strength, and thermal conductivity, and *α* and *β* are the scale factors of *A* and *B*, respectively. The data of material properties are mapped in a *A*–*B* chart. The data can be of different kinds of materials or of the same material from different places or periods, which depends on the given objectives. The data of the same material or of the material from the same place are grouped and colored together for the convenience of identification and comparison.

### 2.4. Ranking and Evaluation

The materials are ranked by contour lines of the performance index in the form of Equation (3), for example,

(4) $$\frac{A^{\alpha }}{B^{\beta }}=C,$$

which is usually expressed in the following logarithmic form:

(5) $$\mathrm{Log}\left(A\right)=\frac{\beta }{\alpha }\mathrm{Log}\left(B\right)+\frac{1}{\alpha }\mathrm{Log}\left(C\right).$$

By plotting the dashed contour lines of the performance index, the data can be quickly ranked and easily read for the typical situations. Usually in a given geological case, the optimal materials are those with the maximum or the minimum value of *M*. This provides a guideline for the comparison among different geomaterials and also gives a quick identification for the given geological event. Several cases for the application of Ashby plots are discussed in the next section for the evaluation of geomaterials in fossil energy exploitation and geological hazard prevention.

## 3. Applications and Results

### 3.1. Coal-Gas Outburst and Coal Seam Roof Instability

**Figure 1:**
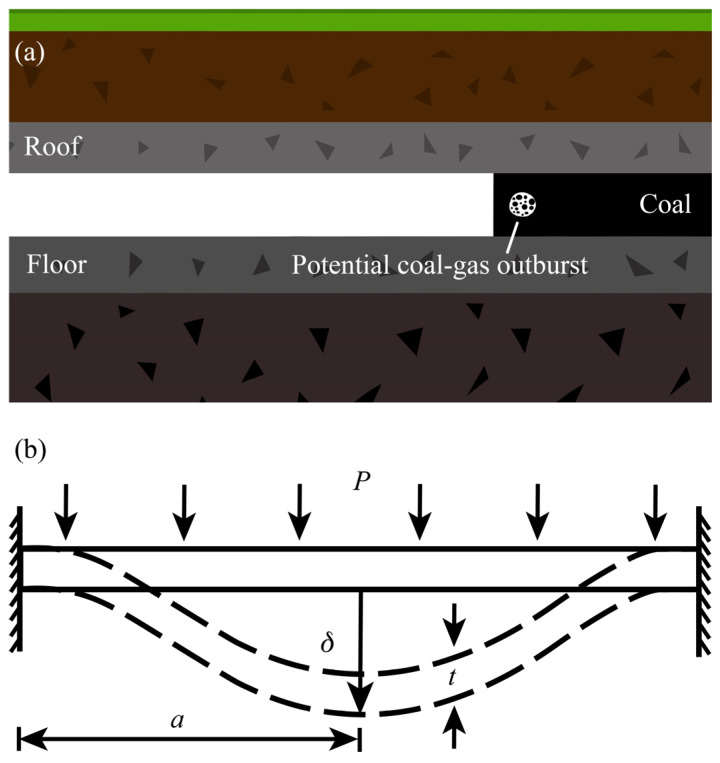
(**a**) Schematic of the roadway with the potential coal-gas outburst in the coal seam. (**b**) Schematic of the deflection of roof and coal wall, where *P* is the pressure caused by rock gravity or gas congregation, *t* is the thickness, *δ* is the deflection in the middle of the plate, and *a* is the half-length of the plate.

Coal-gas outburst and fall of coal seam roof are considered to be the two most significant hazards in the exploitation of coal mines. These two hazards usually cause equipment damage, blockage of emergency escape route, and disruption of ventilation, which result in further casualties and economic losses [2]. Figure 1a shows the inner geological structure near the coal seam roadway in working condition. The roof, which is a rock stratum, becomes unstable and then falls under the pressure caused by gravity of the rocks in upper strata or other man-made stresses. Furthermore, with the tunneling of the coal digger and the congregation of coalbed gas, the risk of coal-gas outburst increases. However, if the coal and the roof show significant deformation before the completed break, workers will be promptly warned to take action or evacuate.

The coal roof and the coal wall in front of the coalbed methane accumulation area are modelled as thin plates with the edge clamped, as shown in Figure 1b. The plate deflects under the pressure caused by rock gravity (for coal roof) and gas congregation (for coal wall). The deflection reaches its maximum value at the center of the plate, that is:

(6) $$\delta =\frac{C_{1}Pa^{4}\left(1-\nu^{2}\right)}{Et^{3}},$$

where *E* is the Young’s modulus, *ν* is the Poisson’s ratio, and *C*_1_ is a constant depending on boundary conditions. For brittle materials such as rocks and coals, the plate deforms elastically until the tensile stress inside the plate reaches the tensile strength *σ*_t_. The maximum tensile stress in the plate caused by the pressure is as follows:

(7) $$\sigma_{t}=C_{2}P\frac{a^{2}}{t^{2}},$$

where *C*_2_ is a constant depending on boundary conditions. Finally, the maximum deflection that the plate can sustain before fracture is derived by eliminating *t* between Equations (6) and (7):

(8) $$\delta_{m}=\frac{C_{1}}{C_{2}^{3/2}}\left(\frac{a}{P^{1/2}}\right)\left[\frac{\sigma_{t}^{3/2}}{E}\left(1-\nu^{2}\right)\right].$$

The rocks and coals which can sustain larger deformation are more stable and have a lower risk of roof fall and coal-gas outburst. That is to say, ideal materials in the cases of roof fall and coal-gas outburst are those having a large value for the performance index:

(9) $$M_{1}=\frac{\sigma_{t}^{3/2}}{E}.$$

**Figure 2:**
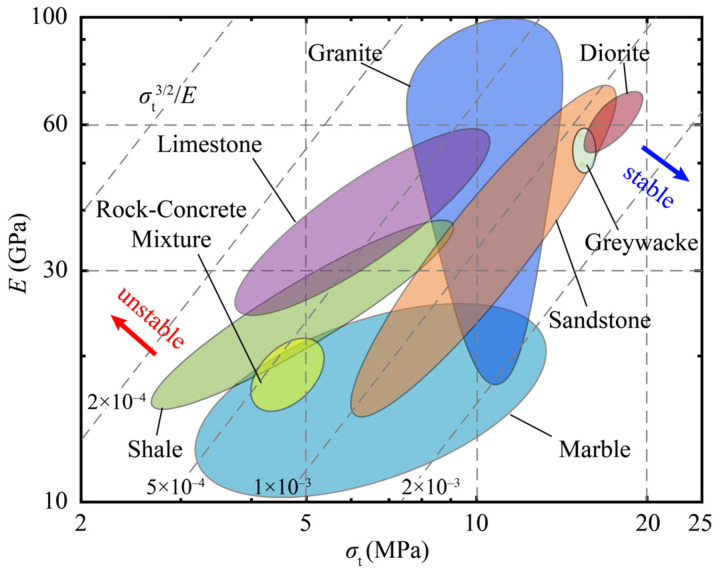
Stability of rocks for coal roof.

Figure 2 shows the Young’s modulus plotted versus the tensile strength, which represents the stability of rock materials in the case of roof fall, according to Equation (9). The performance index acts as the evaluation guideline and is plotted on the chart as broken diagonal contours. Different kinds of rocks are plotted in the chart with each kind of rock in a group. The data of 146 specimens used in the chart are all from the literature [19,20,21,22,23,24,25], in which the Young’s modulus and the tensile strength are derived from laboratory experiments. Rocks towards the top right have a high value of strength and elasticity and therefore can sustain a higher value of stress. At the same time, rocks at the bottom right side have a high value for the performance index, *M*_1_, and can therefore sustain a higher value of deformation. When the above two factors are combined, the direction along which the rocks are more stable as coal roofs is pointed out with the blue arrow in Figure 2. In the same way, the direction along which the coal-roof rocks are more unstable is pointed out with the red arrow in Figure 2. Under this rule of evaluation, diorite is in particular the most stable one among the rocks in this study and shale is the most unstable one as coal roof.

**Figure 3:**
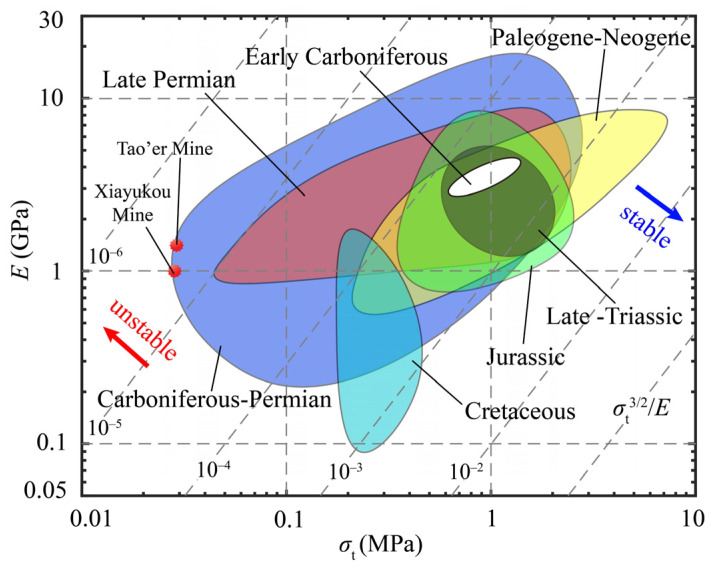
Stability of coals formed in different periods in China.

In the same way, the Young’s modulus versus the tensile strength is plotted in Figure 3 to show the stability of coals in China formed in different periods under the pressure of congregated coalbed gas. All data in Figure 3 are from the literature, [26] in which the data are collected from more than 100 mines in China and are divided into groups by formation periods. The performance index of coals in the chart varies from ~10^−6^ to ~10^−3^, which has a wider range compared with that of rocks plotted in Figure 2. This indicates that there are considerable parts of coals that are more unstable than rocks. For example, parts of coals formed in the Carboniferous-Permian period and the late Permian period have performance index values lower than 10^−4^, which have the high risk of coal-gas outburst under the pressure of congregated coalbed gas. As evidence, coal-gas outbursts occurred in the coals from the Tao’er mine in Hebei province, China, and the Xiayukou mine in Shanxi province, China, in 2007 and 2011, respectively. These coals were both formed in the Carboniferous-Permian period, with both having low tensile strength and a low performance index [26]. In contrast, the coals formed in other periods have a higher performance index and therefore have a lower risk of coal-gas outburst.

In the field, the data of the tensile strength and Young’s modulus can be tested and plotted into the chart to make a further comparison with other geomaterials. In addition, in a typical coal mine, the data derived from different positions can be plotted in a chart to make a parallel comparison to find out potentially dangerous points.

### 3.2. Brittleness of Reservoir Rocks

Brittleness is widely used to characterize the ability of reservoir rocks to form fractures in unconventional energy development, especially in unconventional shale gas and oil exploitation. A large-scale fracture network is more easily formed in reservoirs with high brittleness to improve the permeability and increase the output [27,28]. Therefore, high-brittleness reservoirs are considered as good fracturing candidates. There have been various brittleness indices defined by different researchers to evaluate the brittleness of reservoirs. Jin et al. [29] and Zhang et al. [1] have made detailed reviews of these brittleness indices. Here we introduce a new performance index to evaluate the brittleness of rocks and further express it with the Ashby plot.

Linear elastic fracture mechanics indicates that the stress at a crack tip is singular, which cannot occur in real materials [30]. The Dugdale–Barenblatt model [31,32] solved this problem by assuming there is a yielding, or breakdown, region at the crack tip, named the process zone. The size of the process zone reflects the failure features. Materials with a large process zone in the vicinity of the crack tip, like metals, fail with ductile fractures, whereas other materials that have a small process zone, like ceramics, fail with brittle fractures [9]. In the Dugdale–Barenblatt model, the radius of the process zone is as follows:

(10) $$r_{p}=C_{3}{\left(\frac{K_{\mathrm{IC}}}{\sigma_{c}}\right)}^{2},$$

where *C*_3_ is a constant depending on stress conditions, *K*_IC_ is the fracture toughness, and *σ*_c_ is the cohesive stress. For rock materials, the process zone is a zone where microcracks initiate and propagate, which can often be observed as a mineralized halo for joints and dikes [33]. The radius of the process zone is widely considered to bound the region where the tensile strength *σ*_t_ of the rock is exceeded [34,35]. Therefore, we derived the radius of the process zone by equaling the cohesive stress with the tensile strength in Equation (10) and it is expressed as follows:

(11) $$r_{p}=C_{3}{\left(\frac{K_{\mathrm{IC}}}{\sigma_{t}}\right)}^{2}.$$

A smaller process zone means that the material is more brittle, which indicates that the brittleness of reservoir rocks can be measured by the ratio of the fracture toughness and the tensile strength. Brittle rocks have a low value for the performance index:

(12) $$M_{2}=\frac{K_{\mathrm{IC}}}{\sigma_{t}}.$$

**Figure 4:**
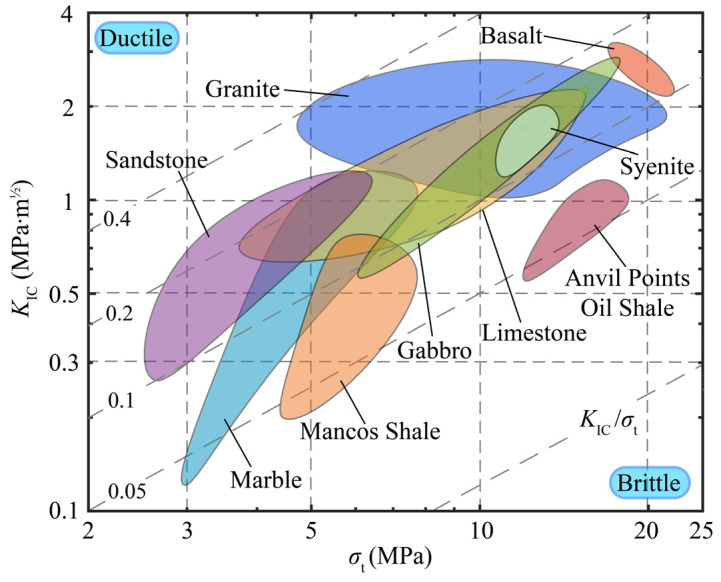
Brittleness of reservoir rocks.

The fracture toughness versus the tensile strength is plotted in Figure 4 to show the evaluation of the brittleness among nine types of rocks. All data in this chart are derived from the literature [19,21,23,24,25,36,37,38]. The ratio of the two mechanical properties varies from 0.05 to 0.4 with the ductility of rocks becoming stronger. The chart demonstrates that the oil shale from Anvil Points, the Mancos shale, and a part of the marble are the most brittle rocks among the nine parallel rocks. In contrast, some of the granites with high toughness and low tensile strength are more ductile when fractured, which means that it is more difficult for the fracture network to be formed in these rocks. More data from the laboratory or the field can be filled into the chart to make a comparison among the reservoirs. This method can also be used to find the best drilling point in a given field to achieve the optimal fracturing effect.

### 3.3. Rock Stability in Landslides

**Figure 5:**
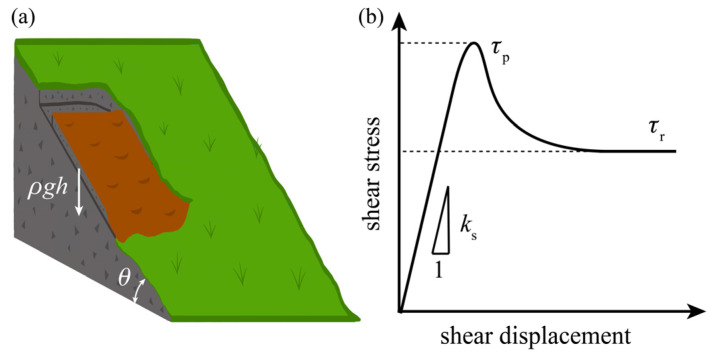
(**a**) Schematic of a land weighting *ρgh* slide along the slope with the incline angle of *θ*; (**b**) typical shear stress–displacement behavior of rocks in direct shear test, where *τ*_p_ is the peak shear strength or the driving strength, *τ*_r_ is the residual shear strength, and the initial slope of the curve, *K*_s_, is the shear stiffness.

As shown in Figure 5a, by considering the land on a slope with an incline angle of *θ*, the shear stress on the contact interface is as follows:

(13) $$\tau =\frac{\rho gh\sin\theta }{S},$$

where *ρ* is the density of the land rock, *g* is the gravitational acceleration, *h* is the thickness of the land, and *S* is the contact area. When the shear stress on the interface or the joint exceeds the peak shear strength, *τ*_p_ (as shown in Figure 5b), the initial landslide occurs and then its sudden shock results in a subsequent rotational slump, after which a series of additional rotational landslides follow until the rocks reaches a stable configuration [39]. This indicates that the development of large landslides begins with a shear slide, which is also the induction factor for the release of slab avalanche [40]. Therefore, we define the ratio of the shear stress and the peak shear strength of the interface to be the danger coefficient as follows:

(14) $$D=\frac{\tau }{\tau_{p}}=\frac{\rho gh\sin\theta }{\tau_{p}S}.$$

The initial landslide occurs when the coefficient *D* ≥ 1. Natural conditions also affect the initial behavior of landslides. Rain increases the density of the rocks and further increases the shear stress on the contact interfaces as well as decreases the shear strength of the rock interfaces. Weathering weakens the shear strength of the interfaces. These will lead to a rise in the danger coefficient and an increase in the risk of landslide. Therefore, the stable rocks in the case of landslide are those with a low value of the following:

(15) $$M_{3}=\frac{\rho }{\tau_{p}}.$$

**Figure 6:**
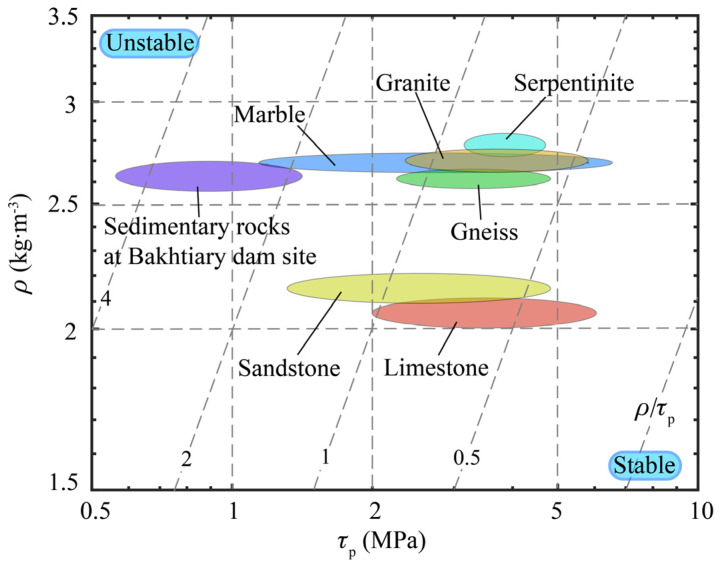
Stability of rock joints in landslides.

The density versus the peak shear strength is plotted in Figure 6 to show the stability of rock joints facing the danger of landslide. The index *M*_3_ is plotted as broken contour lines in the chart. The data in this chart are all from the literature [41,42]. Sedimentary rock joints at the Bakhtiary dam site, Lorestan Province, Iran, are compared with joints of sandstone (from Fribourg, Switzerland), limestone (from Cote d’Or, France), marble (from Apuane Alps, northern Tuscany, Italy), granite (from Tarn, Occitanie region, France), serpentinite (from Valtellina, Italy), and gneiss (from Erstfield, Switzerland). The rock joints towards the bottom right of the chart have low values of *M*_3_ and are therefore stable in the case of a landslide. In contrast, the rock joints towards the top left are more unstable. As a result of the comparison, sedimentary rock joints at the Bakhtiary dam site face a higher risk of landslide than the rock joints from other places listed previously.

### 3.4. Fracture Energy of Geological Faults

The formation of a geological fault is a long timescale process and reflects the cumulative action of historical earthquakes. Faults commonly initiate and grow in weak planes such as rock joints, bedding planes, veins, and interfaces between different sedimentary rocks in the upper crust [35,43]. The frictional ruptures in the geological faults are found to experience significant stress drops (as shown in Figure 5b) and show crack-like behaviors in the initiation and propagation processes [44,45,46]. The Dugdale–Barenblatt model is used to describe and analyze these quasi-static shear cracks with friction [47], in which the maximum half-opening of the crack in the process zone is given as follows:

(16) $$d=\frac{2\left(1-\nu \right)L\sigma_{y}}{\pi \mu }f,$$

where *L* is the length of the crack, *μ* is the shear modulus, *σ*_y_ is the yield strength, and *f* is a dimensionless coefficient depending on the ratio of the breakdown zone length and the crack length. With the assumption that the stress drop, *τ*_p_ − τ_r_, is a constant for a typical fault, the energy release rate (the fracture energy per unit length) of the fault is derived as the following [35]:

(17) $$G=\left(\tau_{p}-\tau_{s}\right)\cdot d=\frac{2\left(1-\nu \right)L{\left(\tau_{p}-\tau_{s}\right)}^{2}}{\pi \mu }f,$$

where the stress drop is performed as the yield strength, *σ*_y_. This equation is effective for the faults formed in intact rocks but not suitable for the rock joints and interfaces between different rocks. The reason is that the shear modulus, *μ*, is invalid in these situations. Therefore, we further expand the form of Equation (17) to be as follows:

(18) $$G=\frac{2\left(1-\nu \right){\left(\tau_{p}-\tau_{s}\right)}^{2}}{K_{s}}f,$$

where *K*_s_ is the shear stiffness of the rock joints and interfaces and *K*_s_ = *μ*/*L* for the intact rocks. Considering that the shear stiffness is the initial slope in the stress–displacement curve (as shown in Figure 5b) of the direct shear experiments, the fracture energy expressed in Equation (18) can not only express the frictional behavior of rock joints and interfaces but can also be tested and calculated using direct shear experiments in the laboratory. Equation (18) indicates that the stable rock structures facing energy fluxes such as earthquakes are those having a high value for the performance index, which is given as follows:

(19) $$M_{4}=\frac{{\left(\tau_{p}-\tau_{s}\right)}^{2}}{K_{s}}.$$

**Figure 7:**
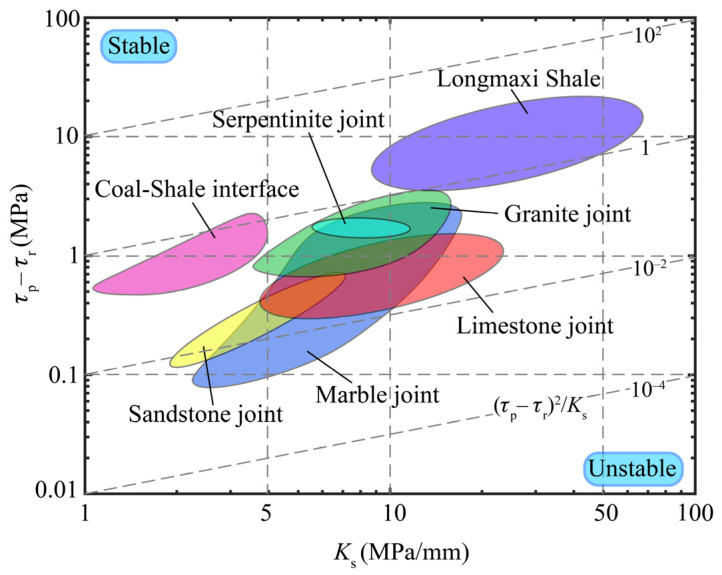
Fracture energy for intact rocks, rock joints, and interfaces.

According to the performance index *M*_4_, the stress drop is plotted against the shear stiffness in Figure 7 to show the evaluation of the stability of intact rocks, rock joints, and interfaces. The data in Figure 7 are all from the literature [41,48,49] and are divided into three main groups—data of intact shales from Longmaxi, Shizhu County, China; data of interfaces of shale and coal from Pocahontas, southern West Virginia, United States; and data of rock joints from the same places listed in Section 3.3. Dashed lines are contours of the performance index, which varies between 10^−4^ and 10^2^. The chart indicates that, among the investigated rocks, the Longmaxi shale (intact rock) is the most difficult to be fractured, which is the same as common sense. The coal–shale interfaces have higher fracture energy than most of the rock joints in this chart, and the marble joints are the most unstable facing the energy fluxes. This chart can be used to evaluate the stability of geomaterials and predict the geological crack paths as long as the data are updated with the data from typical situations.

## 4. Discussion

The preceding case studies illustrate the need for geological engineers to link together comprehensive databases of a large number of different material properties, in order to effectively and quickly evaluate situations involving natural hazards and fossil energy exploitations.

The key factor in drawing the Ashby plots is the performance index. Depending on the role of the performance indices, the Ashby plots in the above applications can be roughly divided into two types. In the first type of Ashby plots, shown in Figure 4, Figure 6 and Figure 7, the performance index is the only factor that directly determines the performance (such as the brittleness and the stability) of geomaterials. Therefore, the ideal materials or the potential dangers appear at the bottom right corner or the top left corner of the plots, where the performance index reaches its maximum or minimum value. In the second type of the Ashby plots, shown in Figure 2 and Figure 3, the performance index is not the only factor that dominates the performance of the materials, because the properties on the axes must be taken into consideration. Therefore, the ideal materials or the potential dangers appear at one side of the top right or the bottom left corner of the plots, where the two properties and the performance index all have high values. The toughness–strength plot (Figure 1a in reference [17]) is of the second type, in which the ideal materials are at the top right corner.

In the cases studies above, the performance indices are all derived by combining two properties. Actually, the performance indices can consist of three or more properties, depending on the factors considered in field applications. The Ashby plots can also be drawn in 3-D form and the axes can be combinations of properties.

Although geological materials may be classified under the same rock type, their mechanical properties may vary greatly depending on intact rock properties, joint patterns, and properties of discontinuities [41,42,50]. This leads to changes in the Ashby plot boundaries in when the number of data increases. This will be overcome when the database is large enough, but there is still a long way to go. However, things are quite different when the problem concerned focuses on a small area or limited specimens. The Ashby plots offer a significant help when engineers want to find the optimum construction site or potential dangers among tens of alternative choices.

## 5. Conclusions

In this study, a scheme was presented to evaluate geological structures from the pure point of view of material properties. This is the first application of Ashby plots in evaluating geomaterials such as rocks and coals in fossil energy development and natural hazard prevention. Four case studies were presented as the applications of the Ashby plots. The stability of coals formed in different periods and rocks as coal roofs were evaluated and compared, respectively. A new brittleness index was theoretically derived to evaluate the fracability of reservoir rocks, which can help in the design of fracturing schemes. The stability of rocks facing the danger of landslides was evaluated. Fracture energy for intact rocks, rock joints, and interfaces to form geological faults were derived and compared. The preceding results show a strong vitality and a prospect of wide application of the Ashby plot in geomaterial evaluation, which may help in geological engineering.
